# Evaluation of Oral Mucosa Elastomers for a 3D Oral Simulation Model

**DOI:** 10.3390/ma18112490

**Published:** 2025-05-26

**Authors:** Joana Mendes, José Manuel Mendes, Lara Coelho, Carlos Aroso, Aritza Brizuela-Velasco, José L. Esteves, Maria Cristina Manzanares-Céspedes

**Affiliations:** 1 UNIPRO—Oral Pathology and Rehabilitation Research Unit, University Institute of Health Sciences (IUCS), Cooperativa de Ensino Superior e Politécnico Universitário—CESPU, 4585-116 Gandra, Portugal; joana.silva.mendes@iucs.cespu.pt (J.M.); lara.coelho@iucs.cespu.pt (L.C.); carlos.ribeiro@iucs.cespu.pt (C.A.); mcmanzanares@ub.edu (M.C.M.-C.); 2 Human Anatomy and Embryology Unit, Faculty of Medicine and Health Sciences, University of Barcelona, 08007 Barcelona, Spain; 3 DENS-ia Research Group, Faculty of Health Sciences, Miguel de Cervantes European University, 47012 Valladolid, Spain; abrizuela@uemc.es; 4 Department of Mechanical Engineering, Faculty of Engineering, University of Porto, 4200-465 Porto, Portugal; jesteves@fe.up.pt

**Keywords:** compressive E-modulus, tensile E-modulus, wettability, elastomers, oral mucosa simulation, shore A hardness

## Abstract

(1) Background: In order to conduct in vitro studies regarding muco-supported dentures, it is necessary to have a simulation model that simulates the oral mucosa, as it is the main influencing factor for their retention and stabilisation. The aim of this study is to perform tensile and wettability tests in three different elastomers to identify the best material for simulating the oral mucosa. (2) Methods: A tensile test was performed according to ISO 527-1 and a compressive test was performed according to ISO 604:2002, at a constant speed of 10 mm/min. The E-modulus was calculated. A wettability test was performed according to ISO 19403-2. Shore A hardness was measured according to ISO 868:2003. All values were compared with the oral mucosa data. (3) Results: Tensile E-modulus calculation revealed no significant difference between Molloplast^®^ B and EXA’lence^TM^ Light Body. The mean drop angle calculation revealed no significant difference between Ufi Gel^®^ SC and Molloplast^®^ B. The compression E-modulus showed significant differences for Ufi Gel^®^ SC and EXA’lence^TM^ Light Body, while Molloplast^®^ B showed no significant deviation. Ufi Gel^®^ SC has a similar Shore A hardness to the other materials. (4) Conclusions: Molloplast^®^ B and Ufi Gel^®^ SC are the most similar elastomers to the alveolar mucosa.

## 1. Introduction

The elderly population is increasing worldwide, especially in developed countries, and this increase is expected to continue over the next 30 years. Consequently, the number of edentulous patients requiring prosthetic treatment is also increasing [1,2,3].

Although fixed rehabilitations are preferred, removable dentures are still used due to several factors, such as economics and anatomical and systemic conditions [2]. Over the past century, both complete and partial removable dentures have been widely used in dentistry to restore function, comfort, and aesthetics to edentulous patients [3,4,5,6].

The oral mucosa is subjected to different levels of external forces, particularly during mastication. In dentate patients, the long axis of the teeth allows for the dissipation of masticatory forces [7]. In edentulous patients wearing removable prostheses, masticatory forces are dissipated though the oral mucosa, distributing the occlusal load to the underlying bone ridge [1,3,6,7]. The oral mucosa behaves mechanically as a viscoelastic material, with time-dependent properties when loaded [1]. The viscoelastic properties of the alveolar ridge mucosa of an edentulous patient play a considerable role, as this is a primary supporting tissue for removable dentures [1,8,9]. When subjected to masticatory forces, the oral mucosa exhibits distinct resistance to deformation under load, comprising a surface epithelial layer and the lamina propria [3]. During mastication, the oral mucosa is subjected to a wide range of mechanical forces, such as hydrodynamic forces, compression, elongation, friction, and shear [1,8]. However, the alveolar ridge is also an important factor in the stress distribution of masticatory forces, as there is an association between the inclination of the alveolar ridge and the position of the load and between the shape of the alveolar ridge and the load location [9].

In order to conduct in vitro studies on removable dentures, it is necessary to have a simulation model that simulates the oral mucosa, as it is the main influencing factor for their retention and stabilisation [1,2,5]. However, there are few studies on simulant materials for the oral mucosa [1,2,10]. Studies have indicated that the elastic properties of the oral mucosa are linear, which means that they have a straight stress–strain response curve with a constant elastic modulus [1] and are highly deformable under compression [3]. The elasticity modulus is also one of the fundamental parameters defining the behaviour of a material, particularly its tendency to deform proportionally to an applied force [3]. The oral mucosa is also considered the most hydrophobic tissue in the human body [11,12]. This is a characteristic of tissues requiring protection against acids or pathogenic organisms, and it plays an important role in several biological processes [12]. The retention of removable prostheses in the oral cavity is achieved through the adaptation of the supporting tissues between the mucosa and the base of the prostheses, through a thin layer of saliva and with atmospheric pressure [2]. Therefore, to choose the best oral mucosa simulant material, tests such as tensile strength (in order to calculate the E-modulus) and wettability tests are necessary to identify the material that mimics the real oral environment of an edentulous patient.

Although in vitro tests related to prosthetic rehabilitations are performed, there is no reliable simulation model of an edentulous patient with oral mucosa that can be used for these studies. Nevertheless, the adhesiveness and behaviour of the oral mucosa is decisive in this type of study. So, the aim of this study is to perform tensile, compression, hardness, and wettability tests on three different elastomers in order to identify the best oral mucosa-simulating material, to subsequently develop a 3D simulation model of a completely edentulous patient for use in laboratory tests on removable and implant-supported dentures.

## 2. Materials and Methods

### 2.1. Materials

All the data of the 3 elastomers tested—their name, composition, brand, type of material, and curing type—are shown in Table 1. The materials were chosen based on their availability on the market, as well as in the literature.

### 2.2. Methods

#### 2.2.1. Specimen Preparation

**Figure 1:**
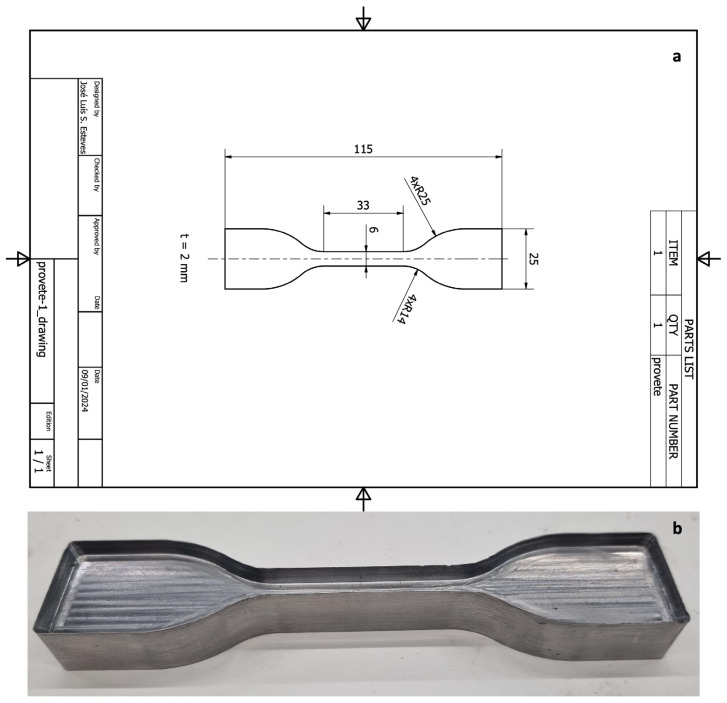
Specimen design: (**a**) technical design of the specimen; (**b**) mould developed to cut the elastomers.

The test specimen spacing and dimensions were prepared as described in ISO 527-3 [13] (Figure 1a). A type 5 specimen was selected for tensile testing. Using the specimen dimensions, a metal mould was produced to cut the elastomers into the correct shape (Figure 1b).

The self-curing elastomers (UFI and EXA) were placed between two plates of polystyrene, with a metal frame made with a 2.5 mm thick aluminium bar to hold the elastomers at the size necessary to obtain the specimens. These materials were distributed in the sheets using a mixing gun (Coltène, Whaledent^®^, Altstätten, Switzerland) with a 1:1 tip, and both leaves were held together with tweezers during the setting time (15 min for both elastomers) at a standard atmosphere 23/50. The elastomer sheets were then removed from the mould.

The heat-cured elastomer (MB) was a paste and not a fluid, so a manual press was used to spread the material between two steel metal plates, with a metal frame made with a 2.5 mm thick aluminium bar to hold the elastomers at the size necessary to obtain the specimens, and polyethylene films were placed so that the material could be removed once it had set. After containing the material, the sheets with the tweezers were placed in an incubator at 100 °C for 2 h. After that time, the elastomer was removed from the mould.

After curing all the materials, each elastomer was placed on top of a polyethylene plate, and the mould that was used to cut the specimens was placed on top of the material, with a thick metal plate on top in order to distribute the forces uniformly. The specimens were cut with the help of a manual press. A digital calliper (Mitutoyo, Manutan Portugal, Carnaxide, Portugal) was used to measure the dimensions in different sites of the specimen. In the same location, both surfaces (upper and bottom) and the middle area of the specimen were measured and the mean value was calculated in order to guarantee the same mean dimensions across all the samples. The size and height of the samples were also confirmed with a digital calliper. Five specimens of each material were produced (*n* = 5/group; total *n* = 15).

#### 2.2.2. Tensile Test

The tensile test was performed in accordance with ISO 527-1 [14]. The specimens were loaded in tension along the longitudinal axis in a testing machine, TIRA test 2705 (TIRA GmbH, Dresden, Germany), with a load cell of 5 kN. The test was performed at a constant speed of 10 mm/min with a force transducer (KAP-S, AST GmbH, Dresden, Germany) until breakage/failure or until the moment when the specimen reached three times the initial length.

For each specimen, the time (s), displacement (mm), and strength (N) were recorded for further analysis. These data were used to calculate the E-modulus in tension (Young’s modulus) using the following equation:

$$E_{t}=\frac{\sigma_{2}-\sigma_{1}}{\varepsilon_{2}-\varepsilon_{1}}$$

where $E_{t}$ is Young’s modulus of elasticity, expressed in megapascals; $\sigma_{1}$ is the stress, in megapascals, measured at the strain value $\varepsilon_{1}$ = 0.05; and $\sigma_{2}$ is the stress, in megapascals, measured at the strain value $\varepsilon_{2}$ = 0.15.

The stress–strain curves of the three elastomers analysed present two zones of distinct behaviour for the E-modulus, with a transition to deformation values in the order of ε = 0.25.

The behaviour is approximately linear both below and above the transition value t, with the use of a secant modulus calculation being a good simplification for reproducing the behaviour of the E-modulus. The E-modulus was determined for the lower deformation zone ε < 0.25 for stress values lower than 0.5 MPa, corresponding to the range of values expected for large cyclic masticatory loads.

#### 2.2.3. Shore Hardness Test

The Shore hardness test was performed using ISO 868:2003 [15]. A 2.5 mm thick sample was manufactured for each elastomer (*n* = 1/group; *n* = 3 in total). A Suter HBA 100-0 type A Shore durometer (Kern & Sohn GmbH, Balingen, Germany) was used. Five measurements were performed by placing the sample horizontally with the durometer in a vertical position. Sufficient pressure was applied to achieve firm contact between the presser foot and the sample at different points on the sample, 6 mm away from each measuring point. The average value of each elastomer was calculated.

#### 2.2.4. Compression Test

The compression test was performed according to ISO 604:2002 [16]. Each material was cut into a cylinder shape with a diameter of 4 mm. Five samples of each material were produced (*n* = 5/group; *n* = 15 in total). The specimens were loaded on the TIRA test 2705 machine (TIRA GmbH, Dresden, Germany) with a load cell of 5 kN. The test was performed at a constant speed of 10 mm/min, with a force transducer (KAP-S, AST GmbH, Dresden, Germany) applying a load from 0 to 250 N. For each specimen, the time (s), displacement (mm), and resistance (N) were recorded for further analysis. These data were used to calculate the compressive E-modulus using the same equation described for the tensile test.

#### 2.2.5. Wettability Test

The wettability test was performed according to ISO 19403-2 [17]. A part of the specimens that was used in the tensile test was also used to perform the wettability test, with a total of 5 specimens for each elastomer (*n* = 5/group; total *n* = 15). The test was performed using a Theta Lite Optical tensimeter TL 100 (Biolin Scientific, Gothenburg, Sweden). The specimen was placed in an adjustable sample holder so that it could be positioned in the lower half of the image and be horizontally aligned. The dosing system was filled with distilled water, and the needle was moved to the upper margin of the image, 4 mm above the specimen. The drop was dosed so that the volume was 2 µL. After this, the image was focused, and the zoom of the contact angle measuring device was adjusted so that the width of the droplet outline occupied two-thirds of the image width. The contact angle measurement was started immediately after dosing using OneAttension 4.0 software (nanoScience Instruments, Phoenix, AZ, USA). The recording started when the droplet was not in contact with the specimen, and, after the droplet was placed on the specimen surface, it was necessary to wait until it stabilised to stop the data recording [18].

Data such as time (s), left contact angle (°), right contact angle (°), mean contact angle (°), volume (µL), and baseline (mm) were recorded for further data processing and a comparison with oral mucosa values.

#### 2.2.6. Statistical Analyses

Data were analysed with R Core Team, version 4.4.1. (R Foundation for Statistical Computing: Vienna, Austria) [19]. Continuous variables are described as means and standard deviations, after checking for skewness and kurtosis within the limits of [−2, 2]. Categorical variables are described as frequencies and percentages. To compare continuous variables by brand, we used a one-way ANOVA (SPSS Inc., Chicago, IL, USA). Partial eta-squared (or just eta-squared in this case) was calculated to measure the effect size following Cohen’s [20] recommendations for low (0.01), moderate (0.06), and high (0.14) effect sizes. After confirming that Cochran’s rules were not met, Fisher’s test was used to assess the association of categorical variables [21].

A one-sample *t*-test was used to specifically compare the mean drop angle with the minimum and maximum targets of 72° and 82°, trying to find the shortest difference.

Significance was determined at *p* < 0.05.

## 3. Results

**Figure 2:**
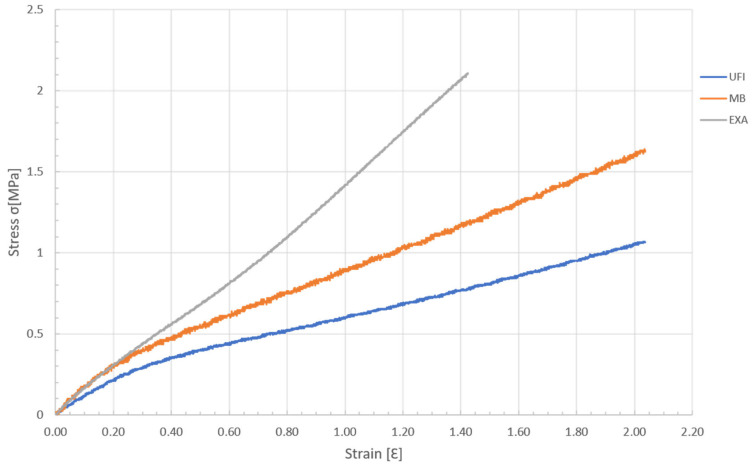
Graph of the stress–strain during the tensile load of the elastomers.

Graphical curves and mean values of the tensile E-modulus of each elastomer are described in Table 2 and Figure 2.

**Figure 3:**
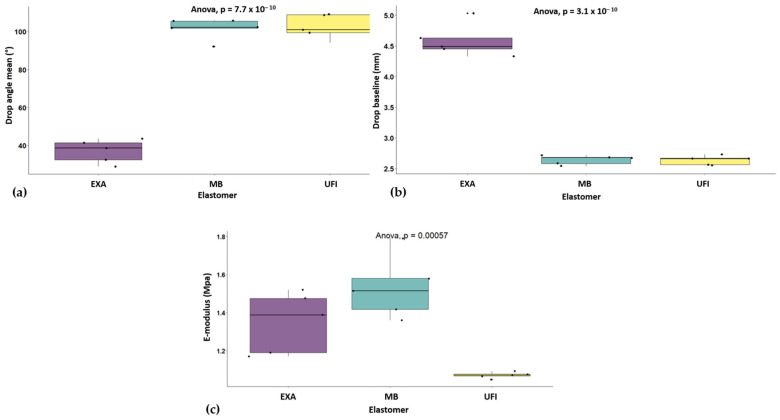
ANOVA analysis: (**a**) drop angle by elastomer; (**b**) drop baseline by elastomer; (**c**) E-modulus by elastomer.

The ANOVA revealed no significant difference in test time between the UFI, EXA, and MB elastomers (F(2,12) = 0.29, *p* = 0.753), with a small effect size (η^2^*_p_* = 0.05). For the mean drop angle, there was a highly significant difference between brands (F(2,12) = 192.40, *p* < 0.001), with a large effect size (η^2^*_p_* = 0.97). Tukey pairwise comparisons showed significant differences between UFI and EXA (*p* < 0.001) and between MB and EXA (*p* < 0.001) but no difference between UFI and MB (*p* = 0.963), with a much lower mean drop angle for EXA than for the other two elastomers (Figure 3a).

Regarding the drop baseline values, there was again a significant difference between the elastomers (F(2,12) = 224.50, *p* < 0.001), with a large effect size (η^2^*_p_* = 0.97). Pairwise comparisons indicated significant differences between UFI and EXA (*p* < 0.001) and between MB and EXA (*p* < 0.001), but no difference between UFI and MB (*p* = 0.999), this time with a higher mean drop baseline for EXA than for the other two elastomers (Figure 3b).

Finally, the E-modulus also showed significant differences (F(2,12) = 14.81, *p* < 0.001), with a large effect size (η^2^*_p_* = 0.71). Tukey comparisons revealed significant differences between UFI and EXA (*p* = 0.018) and between UFI and MB (*p* < 0.001), but no significant difference between MB and EXA (*p* = 0.119). UFI had the lowest mean E-modulus, and MB had the highest (Table 3) (Figure 3c).

An analysis of the proportions of angles ≤ 90° and >90° (Table 4) revealed significant differences between the UFI, EXA, and MB elastomers. UFI and MB had no instances of angles ≤ 90°, with all their data points falling above 90°. In contrast, all of the data points of EXA demonstrated angles ≤ 90°, with none above 90°. Fisher’s test showed a highly significant association between the elastomers and angle category, with *p* < 0.001, indicating that the distribution of angles ≤ 90° and >90° was significantly different between the elastomers, in favour of UFI and MB.

**Figure 4:**
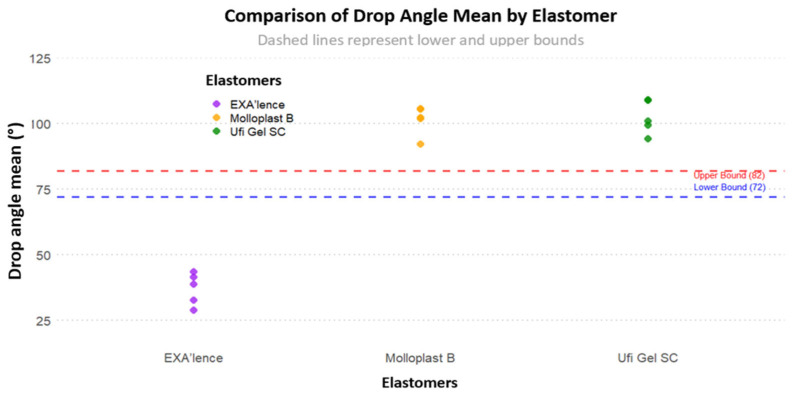
Sample distribution for drop angle mean considering the target of 72–82°.

Figure 4 shows all sample distributions for the drop angle mean considering the target of 72–82°. The drop angle means of all samples were outside the target of 72–82°.

We then compared each elastomer drop angle mean to the targets, trying to find the one with the lowest t-score and therefore closest to non-significance compared to the target (Table 5). The two lowest t-scores were found for UFI for target 82° (t_(4)_ = 7.12, *p* = 0.002) and EXA (t_(4)_ = 7.87 (*p* = 0.001).

Table 6 presents a sample of the *t*-test comparisons of the mean compressive E-modulus considering the target of 2.72 MPa [22]. These results showed significant differences for UFI and EXA, while MB showed no significant deviation. UFI presented a mean compression E-modulus of 2.26 MPa (SD = 0.15), 0.46 MPa lower than the target. This difference was statistically significant (t(4) = −6.71, *p* = 0.003). EXA showed a mean compression E-modulus of 4.68 MPa (SD = 0.19), 1.96 MPa higher than the target, with a highly significant result (t(4) = −22.82, *p* < 0.001). MB presented a mean compression E-modulus of 2.71 MPa (SD = 0.13), with an insignificant difference of +0.01 MPa with respect to the target, which was not statistically significant (t(4) = −0.20, *p* = 0.850). These findings indicate that UFI and EXA deviated significantly from the target compression E-modulus value, while MB conformed closely to it, showing no significant difference.

Table 7 presents the results obtained via the Shore hardness test.

## 4. Discussion

Oral mucosal simulation materials are essential to recreate the mechanical behaviour of edentulous patients’ oral mucosa and thus represent its clinical application in in vitro studies. This study aimed to investigate three different elastomers to select the one that can be used in an edentulous simulation model and perform in vitro oral rehabilitation tests. Finally, MB was the material that presented the most similar wettability and E-modulus results to those of the oral mucosa.

The alveolar oral mucosa and its characteristics are very important when carrying out laboratory tests, especially in cases where the oral mucosa directly interferes with the results. An oral mucosa simulant in an edentulous patient has an important impact when studying mucosa-supported dentures, as it affects the stability and retention of these prostheses. It is also important when studying implant-supported dental prostheses and teeth rehabilitation due to the important behaviour of the oral mucosa in the dissipation of masticatory forces [1,2,3,6,10,11]. However, there is a lack of information regarding alveolar oral mucosa simulation materials that have the same characteristics as the human oral mucosa [1,2,9,11].

The simulant material must have the same hydrophobic characteristics, dimension stability, and resilience as the human oral mucosa [1,2,10,11,23]. Elastomers are a group of chemically or physically cross-linked polymers that have high dimension stability, hydrophobicity, and resilience [23,24]. This study focused on three different elastomers: MB, EXA, and UFI. These elastomers were selected considering their characteristics and results in previous studies [1,11,25]. Elastomers are widely used as simulation material as well as for fabricating maxillofacial prostheses. Hatamleh et al. [26] performed an evaluation of different maxillofacial elastomers. While they [26] attempted to test the bond strength to the substrate, and thus their results cannot be used to compare our findings, a tensile test was performed in their evaluation and one of the conclusions was that the elastomers had sufficient properties as maxillofacial materials, validating the use of elastomers to simulate the oral mucosa. Wieckiewicz et al. [27] conducted a study evaluating silicone interocclusal recoded materials. Elastomers are one of the materials used for occlusal recoding. As in our study, tensile tests were also performed to evaluate the elastic properties of these materials.

Both Al-Kaisy N [11] and Mei H et al. [12] referred to the oral mucosa as the most hydrophobic tissue of the human body; thus, in order to account for that characteristic, each elastomer studied was classified as a hydrophobic or hydrophilic material. During the wettability tests, surface hydrophobicity can be categorised as hydrophilic (contact angle < 90°), hydrophobic (contact angle > 90°), over-hydrophobic (120° < contact angle < 150°), and superhydrophobic (150° < contact angle < 180°, sliding angle < 10°) [28]. Both the MB and UFI elastomers presented a contact angle over 90°, confirming their hydrophobicity. EXA presented a contact angle of less than 90°, characteristic of a hydrophilic material.

Al-Kaisy N [11] also reported that the attached gingiva in the central incisor area has a contact angle between 72° and 82°. Mei et al. [12] also presented a similar range for the contact angle in the attached gingiva in their study. Some studies have analysed the contact angle of different parts of the human body. In the oral cavity, most studies have focused on the wettability of the tongue. However, the hydrophobicity of the tongue is very different from that of the oral mucosa [29]. As no values for the alveolar oral mucosa were found in the literature, the contact angles of the different elastomers were compared with those presented by Al-Kaisy N [11].

The contact angles of MB and UFI were above the range value of 72–82°. However, they were closer to this range than EXA, which presented a contact angle much lower than expected. Thus, in terms of the wettability and contact angle evaluation of the three elastomers, MB and UFI presented hydrophobic behaviour and were closest to the values of the attached gingiva in the area of the central incisors.

The elastic modulus of each elastomer was calculated from the results obtained in the tensile tests. When compared with each other, significant differences were found between UFI and the other two elastomers. However, there were no significant differences between the remaining elastomers (MB and EXA). When comparing the E-modulus with the values found by Choi et al. [7] in their study on cadavers, it should be noted that they used a constant strain rate of 20 mm/min in the tests, as opposed to the value of 10 mm/min used in our analysis, resulting in lower E-modulus values in the present study. We found that the studied elastomers (MB E-modulus: 1.5324 (±0.1680) MPa; UFI E-modulus: 1.0723 (±0.0159) MPa; EXA E-modulus: 1.3484 (±0.1607) MPa) presented values closer to those of the attached gingiva (E-modulus: 8.33 (±5.78) MPa) than those of the buccal mucosa (E-modulus: 37.36 (±17.36) MPa) or hard palate (E-modulus: 18.13 (±4.51) MPa).

Although this study followed ISO standards for tensile testing, the results are not in accordance with the E-modulus calculated by Choi et al. [1] for MB and UFI, two of the eleven tested by the authors. In the study by Choi et al. [1], MB presented an elastic modulus of 0.89 (±0.16) MPa, and UFI presented an elastic modulus of 0.58 (±0.10) MPa. The selection of different specimen types may be a reason for this difference. In our study, a type 5 specimen with a constant speed of 10 mm/min was used; however, Choi et al. [1] used a type 1B specimen with a constant speed of 1 mm/min.

Goktas et al. [30] studied the in vitro behaviour of porcine tissues and performed tensile tests in different regions of oral soft tissues. The results also do not agree with those of the present study, as the inserted gingiva presents an E-modulus of 18.83 (±5.98) MPa for the lingual region and 19.75 (±6.20) MPa for the buccal region. These results are more similar to those found by Choi et al. [7] in the human hard palate. This could also be due to the crosshead speed used during the test, as Goktas et al. [30] used a crosshead speed of 5 mm/min.

A Shore A hardness test was performed to classify each elastomer used in the present study. No values were found for the Shore A hardness of alveolar oral mucosa to compare data, but results of each elastomer can be compared with each other. MB was the elastomer with higher Shore A hardness. Compared with the other two, EXA has a closer hardness compared with UFI. According to Mese et al. [31], heat-cured silicone-based materials have better properties and are harder than self-cured materials. In their study, they performed hardness tests on several denture liners, including Molloplast B. MB was found to have a lower value (between 42.28 (±0.89) and 46.16 (±2.15), depending on time). The difference between these values and those presented by MB in our study may be due to sample size. In our study, the elastomer samples had an average thickness of 2.5 mm so that the Shore A hardness values could be compared to the ones measured on oral mucosa in vivo. The thickness of our samples was much smaller than that used in Mese et al. [31], which explains the higher values in our study. Dederichs et al. [32] conceptualised the construction of an in vitro gingival sulcus model to study gingival retraction materials. In their study, they selected a silicone simulation material, Profisil 15 (Kettenbach GmbH & Co. KG, Eschenburg, Germany), because it has a Shore A hardness of 15. They used this value because another study about human soft tissue showed that healthy human soft tissue has a Shore A hardness of 16 to 21. However, this study was on plantar ulcers and did not study oral mucosa, so these values cannot be compared since oral mucosa is harder than foot tissue. However, based on the idea that human soft tissue has a low Shore A hardness, UFI appears the most appropriate.

The compressive E-modulus results were compared with the value used in the study by Lacoste-Ferré et al. [22], where an E-modulus 2.72 MPa of dehydrated oral mucosa was used as a reference, since it is the most representative of the clinical reality. The mean compressive E-modulus of MB was 2.71 (±0.13) MPa, showing no significant difference between dehydrated oral mucosa, unlike UFI (2.26 (±0.15) MPa) and EXA (4.68 (±0.19) MPa), which showed differences between the comparative values. When analysing the data, MB and UFI obtained much closer compressive E-modulus values compared to EXA. Several studies have analysed viscoelastic behaviour of oral mucosa and its dynamic properties [11,22,30]. However, the main objective of this study is to select an ideal artificial material for the oral mucosa that allows for in vitro studies, without permanent deformation, and that recovers its initial geometry for reuse.

Tissue-engineered models of oral mucosa are also available on the market. These 3D organotypic tissue models use normal human cells from healthy donors and allow for the evaluation of cellular and tissue behaviour [33]. Since this type of tissue has low longevity and cannot be adapted to a mandibular model for mechanical testing, this type of oral mucosa simulant should only be used for biological purposes.

After analysing all the test results, especially the hydrophobicity and the compressive modulus of the elastomers, it was determined that EXA is not a good simulant of the alveolar mucosa. However, MB showed significant differences between the compressive modulus and compressive E-modulus of dehydrated oral mucosa. As for MB and UFI, tensile E-modulus showed significant differences in favour of MB. Furthermore, Shore A hardness favours UFI, since it presents a lower value than MB and EXA.

It should also be taken into account that MB is a cured material; it is presented in paste form, and its handling is not easy. In addition, it has very strong adhesiveness, so it requires good isolation. In this respect, UFI is much easier to use and handle.

The main limitation of this study is the tests performed on the elastomers, in which the dynamic compression E-moduli were not studied. This study only focused on preliminary mechanical testing of the three different elastomers to select the one closest to the oral mucosa. The dimensional stability and dynamic compression E-modulus should be calculated once a trustable 3D model is obtained. Another limitation of this study is the lack of information on the mechanical values of the human oral mucosa. There is a lack of information regarding the wettability values of edentulous mucosa, and the values that can be found in the literature focus only on the attached gingiva in dentate patients or on their tongue [12,29]. The same is true for the calculation of tensile E-modulus, as only one article has reference values for this test, which were obtained from cadaver oral mucosa (Choi et al. [7]). Information on the Shore A hardness of oral mucosa is also missing in the literature. Further studies should be conducted to analyse human edentulous mucosa in order to obtain reference values for comparison with the results of the various mechanical tests needed to find the elastomer that can simulate the oral mucosa in in vitro testing.

## 5. Conclusions

Materials that simulate the oral mucosa, such as elastomers, are essential for the development of an edentulous mandibular prototype for laboratory testing. In this study, MB was the elastomer most similar to the alveolar mucosa. UFI also performed favourably, exhibiting a lower Shore A hardness, similar to that of oral mucosa, but a lower E-modulus for tensile and compression testing, which favours MB as a simulation material. However, UFI is an easier product to use in the development of a simulation model. The clinical prospects are to prototype a simulation model of an edentulous patient to enable more reliable laboratory testing and to facilitate improved quality of life for oral rehabilitation patients.

## Figures and Tables

**Table 1:** Dental elastomers used in this study.

Material Type	Material Name	Brand	Composition	Curing Type
Soft denture liner	Molloplast^®^ B (MB)	Dentax, GmbH, Ettlingen, Germany	Dibenzoyl peroxide; benzoyl peroxide; dodecaemthylcyclohexasiloxane	Heat cure
Soft denture liner	Ufi Gel^®^ SC (UFI)	VOCO GmbH, Cuxhaven, Germany	Vinyl polysiloxane	Self-cure
Impression material	EXA’lence^TM^ Light Body (EXA)	GC Corporation, Leuven, Belgium	Vinyl polyether silicone	Self-cure

**Table 2:** Tensile E-modulus values of the different elastomers.

Elastomer	E-Modulus (Young’s Modulus)
Molloplast^®^ B (MB)	1.5 (±0.17) MPa
Ufi Gel^®^ SC (UFI)	1.1 (±0.02) MPa
EXA’lence^TM^ Light Body (EXA)	1.3 (±0.16) MPa

**Table 3:** ANOVA comparisons of elastomers.

	Ufi Gel^®^ SC	EXA’lence^TM^	Molloplast^®^ B	F-Test	Tukey Pairwise Comparisons
Test time (s)	9.98 ± 0.03	9.98 ± 0.03	9.97 ± 0.03	F_(2,12)_ = 0.29, *p* = 0.75, η^2^_p_ = 0.05	-
Drop angle mean (°)	102.51 ± 6.44	36.98 ± 6.14	101.50 ± 5.54	F_(2,12)_ = 192.40, *p* < 0.001 ***, η^2^_p_ = 0.97	UFI vs. EXA (*p* < 0.001 ***) MB vs. EXA (*p* < 0.001 ***) UFI vs. MB (*p* = 0.96)
Drop baseline (mm)	2.63 ± 0.08	4.59 ± 0.27	2.64 ± 0.07	F_(2,12)_ = 224.50, *p* < 0.001 ***, η^2^_p_ = 0.97	UFI vs. EXA (*p* < 0.001 ***) MB vs. EXA (*p* < 0.001 ***) UFI vs. MB (*p* = 0.99)
Tensile E-modulus (MPa)	1.07 ± 0.02	1.35 ± 0.16	1.53 ± 0.17	F_(2,12)_ = 14.81, *p* < 0.001 ***, η^2^_p_ = 0.71	UFI vs. EXA (*p* = 0.02 *) UFI vs. MB (*p* < 0.001 ***) MB vs. EXA (*p* = 0.12)

ns: non-significant; * *p* < 0.05; *** *p* < 0.001; effect size calculated as partial eta-squared (η^2^_p_) following Cohen’s recommendations for low (0.01), moderate (0.06), and high (0.14) effect sizes [19].

**Table 4:** Comparison for proportions of angles ≤/> 90° in each brand.

	Angle ≤ 90°	Angle > 90°	Fisher’s Test
Ufi Gel^®^ SC	0 (0%)	5 (50.0%)	*p* < 0.001 ***
EXA’lence^TM^ Light Body	5 (50.0%)	0 (0%)	
Molloplast^®^ B	0 (0%)	5 (50.0%)	

*** *p* < 0.001; Fisher’s statistic was computed to measure the association between brand and angle because Cochran’s rules were not met [19].

**Table 5:** One-sample *t*-test comparison of drop angle mean considering the closest threshold.

	Target 72°	Target 82°
Ufi Gel SC	t_(4)_ = 10.59 (*p* < 0.001 ***)	t_(4)_ = 7.12 (*p* = 0.002 **)
EXA’lence^TM^ Light Body	t_(4)_ = −12.75 (*p* < 0.001 ***)	t_(4)_ = −16.36 (*p* < 0.001 ***)
Molloplast^®^ B	t_(4)_ = 11.91 (*p* < 0.001 ***)	t_(4)_ = 7.87 (*p* = 0.001 **)

** *p* < 0.01; *** *p* < 0.001.

**Table 6:** One-sample *t*-test comparisons of the mean compression E-modulus considering the target of 2.72 MPa [22].

	Mean (SD)	Mean Difference to Target of 2.72 MPa	Target of 2.72 MPa
Ufi Gel SC	2.26 (±0.15)	+0.46	t_(4)_ = −6.71 (*p* = 0.003 **)
EXA’lence	4.68 (±0.19)	−1.96	t_(4)_ = −22.82 (*p* < 0.001 ***)
Molloplast B	2.71 (±0.13)	+0.01	t_(4)_ = −0.20 (*p* = 0.850)

** *p* < 0.01; *** *p* < 0.001.

**Table 7:** Shore A hardness for each elastomer.

	Ufi Gel SC	EXA’lence	Molloplast B
Test 1	51	64	68
Test 2	50	63	69
Test 3	50	62	70
Test 4	52	64	72
Test 5	51	62	69
Mean (SD)	50.8 (±0.7)	63 (±0.9)	69.6 (±1.4)

## Data Availability

The original contributions presented in this study are included in the article. Further inquiries can be directed to the corresponding author.
